# Old Linen and Calico

**Published:** 1887-10-15

**Authors:** Alice Dannatt

**Affiliations:** Hospital Matron and Nurse Superintendent, Holydyke, Barton-on-Humber


### OLD LINEN AND CALICO.

If the Secretary to the Great Northern Central Hospital, etc., who in last week's issue of The Hospital says his stock of old linen is exhausted, would, in his appeals for old linen, insert "calico," he would receive plenty of well-worn old calico, which is pretty nearly as good as old linen, and all that we can expect to get nowadays, because linen is very little worn. The general public think that only linen will do. Old calico is a better substitute for old linen than cotton lint is for real lint. Cotton lint, as no doubt you know, is in general use in all hospitals. May I ask why lint is made more and more fluffy? I suppose the idea is to make it softer. The fluff makes the lint stick, and if I may use the term, eat into wounds much more than it ought to do, and often, however carefully the dressing is taken off, too much irritation is caused. Might not old linen (calico), clean of course, and carbolised, be used much oftener than it is for dressings, instead of cotton lint?

Alice Dannatt,
Hospital Matron and Nurse Superintendent.

Holydyke, Barton-on-Humber, Oct. 5, 1887.

